# Influence of pilot and small trials in meta-analyses of behavioral interventions: a meta-epidemiological study

**DOI:** 10.1186/s13643-023-02184-7

**Published:** 2023-02-18

**Authors:** Michael W. Beets, R. Glenn Weaver, John P. A. Ioannidis, Christopher D. Pfledderer, Alexis Jones, Lauren von Klinggraeff, Bridget Armstrong

**Affiliations:** 1 Arnold School of Public Health, University of South Carolina, SC Columbia, USA; 2 Department of Medicine, Stanford University, Stanford, CA USA; 3 Department of Health Research and Policy, Stanford University, Stanford, CA USA; 4 Department of Biomedical Data Science, Stanford University, Stanford, CA USA; 5 Department of Statistics, Stanford University, Stanford, CA USA; 6 Departments of Medicine, of Health Research and Policy, of Biomedical Data Science, and of Statistics, Meta-Research Innovation Center at Stanford (METRICS), Stanford University, Stanford, CA USA

**Keywords:** Recommendations, Public health, Behavioral science, Preliminary studies

## Abstract

**Background:**

Pilot/feasibility or studies with small sample sizes may be associated with inflated effects. This study explores the vibration of effect sizes (VoE) in meta-analyses when considering different inclusion criteria based upon sample size or pilot/feasibility status.

**Methods:**

Searches were to identify systematic reviews that conducted meta-analyses of behavioral interventions on topics related to the prevention/treatment of childhood obesity from January 2016 to October 2019. The computed summary effect sizes (ES) were extracted from each meta-analysis. Individual studies included in the meta-analyses were classified into one of the following four categories: self-identified pilot/feasibility studies or based upon sample size but not a pilot/feasibility study (*N* ≤ 100, *N* > 100, and *N* > 370 the upper 75th of sample size). The VoE was defined as the absolute difference (ABS) between the re-estimations of summary ES restricted to study classifications compared to the originally reported summary ES. Concordance (kappa) of statistical significance of summary ES between the four categories of studies was assessed. Fixed and random effects models and meta-regressions were estimated. Three case studies are presented to illustrate the impact of including pilot/feasibility and *N* ≤ 100 studies on the estimated summary ES.

**Results:**

A total of 1602 effect sizes, representing 145 reported summary ES, were extracted from 48 meta-analyses containing 603 unique studies (avg. 22 studies per meta-analysis, range 2–108) and included 227,217 participants. Pilot/feasibility and *N* ≤ 100 studies comprised 22% (0–58%) and 21% (0–83%) of studies included in the meta-analyses. Meta-regression indicated the ABS between the re-estimated and original summary ES where summary ES ranged from 0.20 to 0.46 depending on the proportion of studies comprising the original ES were either mostly small (e.g., *N* ≤ 100) or mostly large (*N* > 370). Concordance was low when removing both pilot/feasibility and *N* ≤ 100 studies (kappa = 0.53) and restricting analyses only to the largest studies (*N* > 370, kappa = 0.35), with 20% and 26% of the originally reported statistically significant ES rendered non-significant. Reanalysis of the three case study meta-analyses resulted in the re-estimated ES rendered either non-significant or half of the originally reported ES.

**Conclusions:**

When meta-analyses of behavioral interventions include a substantial proportion of both pilot/feasibility and *N* ≤ 100 studies, summary ES can be affected markedly and should be interpreted with caution.

**Supplementary Information:**

The online version contains supplementary material available at 10.1186/s13643-023-02184-7.

## Background

Public health recommendations should be based upon the most rigorous scientific evidence available [1]. In the behavioral sciences, public health recommendations often originate from well-defined systematic reviews where multiple studies of a given topic are synthesized via meta-analytical techniques [2, 3]. Findings from these reviews often inform evidence-based decision making [2]. Although no definitive guidelines exist for the types of studies required for evidence-based decision-making, in the behavioral sciences, where intervention delivery and impact can be influenced by a myriad of factors, an important consideration is whether a recommendation is based upon trials that are representative of anticipated conditions, and subsequently, exhibit effects more consistent with what is expected under real-world delivery conditions [4].

In clinical research, larger trials are often associated with smaller effects. Evidence from larger trials is widely believed to be more consistent with the to-be-expected true effect than the effects from smaller trials [5]. Conversely, effects from smaller clinical trials are often larger and more heterogeneous and are thus associated with more uncertainty. Despite this, the vast majority of clinical research is conducted on “small” samples (~ 100 participants or fewer) [6] and over two-thirds of all meta-analyses in the Cochrane reviews are based solely on underpowered trials [7]. In the behavioral sciences, as with clinical research, it is important to consider whether effects from smaller trials may be affecting substantially the results of meta-analyses and subsequently also influence public health recommendations.

Evidence demonstrates that effects produced from smaller trials and explanatory designs may fail to translate when evaluated in larger studies delivered under more pragmatic, real-world conditions [8, 9]. In the behavioral sciences, conducting smaller trials of an intervention is a common practice and routinely occurs during the early-stage of developing and testing an intervention in order to evaluate feasibility markers associated with conducting a larger, more well-powered definitive trial [10]. Collectively referred to as pilot/feasibility trials, such trials are becoming increasingly common in the published literature and are recognized as a necessary prerequisite for the receipt of funding for a larger trial [11]. Pilot/feasibility trials are often smaller versions of the intended larger, more well-powered trial, yet are typically not designed with the primary purpose of demonstrating efficacy, but rather to establish that key components of the trial processes work together successfully. For these reasons pilot/feasibility studies are often underpowered and not designed to yield effects expected in the larger trial [12]. Although pilot/feasibility studies are commonly conducted with smaller samples, given their developmental nature and the uncertainties of the intervention processes evaluated, they are conceptually distinct from studies of similar size that are not labeled as a pilot/feasibility trial.

This study aims to examine the impact of including smaller and pilot/feasibility trials of behavioral interventions in summary effect size estimates in meta-analysis. Further, this study explores whether public health recommendations might be impacted by the inclusion of smaller and pilot/feasibility trials in meta-analyses. In this paper we explore these issues in a sample of meta-analyses on topics related to behavioral interventions targeting childhood obesity. We present evidence on the prevalence of smaller and pilot/feasibility trials and the impact of excluding smaller or pilot/feasibility trials in relation to the reported summary meta-analytic effects. Additionally, we present case studies illustrating the impact of smaller and pilot/feasibility studies on conclusions drawn from meta-analyses used to inform public health recommendations.

## Methods

### Study design

A meta-epidemiological review was conducted to identify published systematic reviews that conducted a meta-analysis (referred to throughout the remainder of this study as “meta-analyses”) that met our inclusion criteria (see below), with all reviews of database updated and finalized by October 31, 2019. The procedures for identifying the set of systematic reviews that conducted a meta-analysis and outcomes are reported according to the PRISMA (Preferred Reporting Items for Systematic review and Meta-Analysis) statement [13] (Additional file 1).

### Data sources

A comprehensive literature search to identify systematic reviews that conducted a meta-analysis was conducted across the following databases: PubMed/MEDLINE; Embase/Elsevier; EBSCOhost, and Web of Science. A combination of MeSH (Medical Subject heading), EMTREE, and free-text terms, and any Boolean operators and variants of terms, as appropriate to the databases, were used to identify eligible publications (Additional file 2). Each search included “systematic” or “meta-analysis” in the title or abstract along with one or more of the following terms for the sample’s age (child, preschool, school, student, youth, and adolescent) and one of the following terms to be identified as a topic area related to childhood obesity (obesity, overweight, physical activity, diet, nutrition, sedentary, screen, diet, fitness, or sports). Two authors (MB and AO) screened and reviewed all articles for inclusion. We restricted our search to meta-analyses published since January 1, 2016.

### Inclusion/exclusion criteria

All meta-analyses were screened for inclusion based upon the following criteria: reported on a child obesity-related topic (see above), included behavioral interventions, presented studies summarized via meta-analytical procedures, and included at least two or more studies. Exclusion criteria included mechanistic feeding or lab-based exercise training studies, institutionalized sample, conference presentation/abstract with no full-length publication, special populations with known sample size limitations (e.g., cerebral palsy, childhood cancer, neuromotor delays, massively obese, disorder sleep), reported clinical outcomes solely (e.g., glucose, blood pressure), or reported only post-hoc comparisons or confounder/effect modifier comparisons. These categories of studies were excluded to ensure only meta-analyses of behavioral interventions were included for analysis. Once all meta-analyses were identified, the reference lists were reviewed and all included studies in the meta-analyses retrieved.

### Data extraction

Data for each meta-analysis article were extracted from the summary effects presented in forest plots. Extracted data from the forest plots included the sample size for each individual study (where presented), and the continuous outcome point estimates, and 95% confidence intervals (95% CI) or standard error, where reported. Where sample sizes of the individual studies were not reported, this was extracted from the original publication. Where individual studies were included in more than one meta-analysis, we extracted all effects assuming independence. The metric of the effects presented (Hedges’ *g*, mean difference in the units of the outcome, or standardized mean difference) was recorded. All summary effects represented in forest plots within each meta-analysis article were translated into standardized mean differences (SMD) for analytical purposes. All effect sizes were corrected for differences in the direction of the scales so that positive effect sizes corresponded to improvements in the intervention group, independent of the original scale’s direction. This correction was performed for simplicity of interpretive purposes so that all effect sizes were presented in the same direction and summarized within and across studies. Two authors (MB and AO) extracted and verified all data from included articles.

### Classifying studies based on sample size and pilot/feasibility status

For our primary analyses, individual studies from the meta-analyses were a priori classified into four categories based upon either sample size or pilot/feasibility designation (i.e., self-identified pilot/feasibility, *N* ≤ 100, *N* > 100, and *N* > 370). The first classification was for pilot/feasibility studies which were defined as those studies that self-identified in the title, abstract, or main body of the publication as being a pilot, feasibility, exploratory, evidentiary, or proof of concept trial. Pilot/feasibility trials were coded separately from the other trials irrespective of the included sample size. For all other studies that did not self-identify as a pilot/feasibility trial, the following three sample size classifications were made. We classified studies according to previously published sample size categories which defined smaller trials as including 100 or fewer total participants (*N* ≤ 100 studies) [6]. We classified the remaining, non-pilot/feasibility trials as *N* > 100 (i.e., excludes both pilot/feasibility and *N* ≤ 100 studies). As a secondary classification among trials with *N* > 100, we separated those trials with the largest 25% of sample sizes according to the distribution of sample sizes presented in studies included in the meta-analyses. For this sample of studies, this corresponded to *N* > 370, and served as our fourth study classification.

### Prevalence of smaller and pilot/feasibility trials in meta-analyses

We explored the prevalence and overlap of trials using network visualization analyses tools. All individual articles (i.e., edges) included in the identified meta-analyses were coded based upon pilot/feasibility study and sample size classifications, the origin meta-analysis publication (i.e., node) and entered into Gephi (v.0.9.2) [14]. We examined the number of unique articles included across meta-analyses by trial sample size and pilot/feasibility classifications, and examined the potential overlap of individual trials included across multiple meta-analyses (whether individual studies are included in more than one meta-analysis).

### Data analyses

#### Influence of trial classification on summary effects from meta-analyses

For the purpose of this study, we defined the vibration of effects (VoE) as the difference between the originally reported summary meta-analytical SMD and the re-estimated summary SMD restricting analyses to trials based upon the classifications as defined above [15]. We represent the VoE using the following two metrics: (1) the absolute difference in the estimated ES between the originally reported summary meta-analytical SMD and the re-estimated summary SMD; and (2) the percent difference in the estimated ES between the originally reported summary meta-analytical SMD and the re-estimated summary SMD by dividing the re-estimated summary SMD by the originally reported summary meta-analytical SMD.

We also examined the influence of the proportion of studies of a given classification within a summary SMD on the VoE as defined as the absolute difference in the SMD using meta-regression. The absolute difference was used to determine the overall deviation (i.e., vibration), regardless of directionality, from the originally reported based upon the re-estimations as the dependent variable and the proportion of studies based on quintiles (i.e., ≤ 20%, 21–40%, 41–60%, 61–80%, > 80% of studies) and entered into the model as dummy coded independent variables for analyses restricted to *N* > 100 and *N* > 370. In all analyses, unless stated otherwise, the *N* > 100 group includes studies with sample sizes greater than 370. In models restricted to pilot/feasibility and *N* ≤ 100 studies, too few (*n* = 3) summary ES were comprised of 80–100% of studies of these classifications. Therefore, a group comprised of > 60% was created and entered into the models along with ≤ 20%, 21–40%, 41–60%. The overlap of individual studies included in more than one meta-analysis was not accounted for in the analyses given the level of inference was on the original and re-estimated summary effect size and not estimates of individual studies. Separate models were run for each of the four study classifications. Each model controlled for the number of articles included in a meta-analysis to account for differences in the total number of studies represented across meta-analyses, with some meta-analyses comprised of very few studies (e.g., < 10) and other comprised of many studies (e.g., ≥ 50). Models were estimated using the *meta* commands in Stata (v.16.1, College Station, TX).

Additionally, we compared the nominal level of statistical significance by comparing the agreement (i.e., level of concordance) between the level of nominal statistical significance (*P* ≤ 0.05 vs. > 0.05) from the re-estimated summary effects and the originally reported meta-analytical effects. Summary effects were classified as either significant or non-significant and the level of concordance evaluated using kappa coefficient. Finally, the association between study classification and precision, defined as 1/SE, was also investigated by comparing the precision of studies based upon the four classifications and the decile of precision. Summary ES were estimated across deciles of precision.

### Case study examples

To illustrate the influence of VoE resulting from including smaller or pilot/feasibility studies in meta-analyses, we identified three US Preventive Services Task Force and the Community Preventive Services Task Force websites for recommendations that met the following criteria: targeted youth (≤ 18 years), focused on a topic area related to childhood obesity (obesity, overweight, physical activity, diet, nutrition, sedentary, screen, diet, fitness), were based upon a meta-analysis and the data in the article were presented in a forest plot to extract the computed SMD and measure of variance for each individual study. For identified meta-analyses, we retrieved the publications of the studies included and categorized them according to our four classifications: self-identified pilot/feasibility, *N* ≤ 100, *N* > 100, and *N* > 370. Using the procedures outlined above, we compared the re-estimated summary SMD vs. the originally reported summary SMD in the identified meta-analyses to identify differences in the estimates and conclusions considering trials of differing size.

All data were entered into comprehensive meta-analysis (v.3.3.07, Englewood, NJ) to calculate the standardized mean difference effect sizes for each reported outcome across all studies. The summary SMD were computed for the originally reported summary SMD and for all comparisons based on study classifications. All analyses were made at the summary SMD level and computed using both fixed-effects estimates and the DerSimonian–Laird random-effects estimates [16]. For instances where the originally reported summary SMD was comprised of studies all the same classification, no comparisons were made (e.g., nine studies in summary SMD and all classified as *N* ≤ 100).

## Results

### Articles selected for the review

**Fig. 1:**
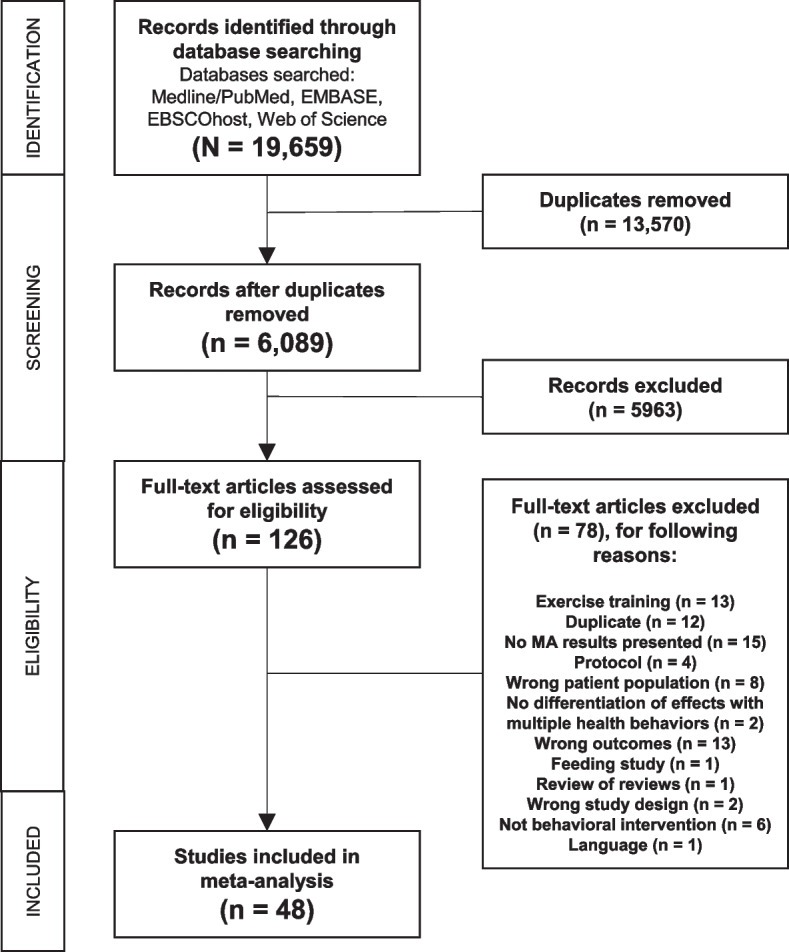
PRISMA diagram for identification of studies to include in the meta-epidemiological review

The PRISMA diagram for the database search and final inclusion of articles is presented in Fig. 1. Overall, a total of 19,659 records were identified, consisting of 6089 unique records. Of these, 126 were deemed potentially relevant upon title/abstract screening. A final set of 48 meta-analyses [17–64] meeting all relevant criteria were retained and used for analyses. The 48 meta-analysis articles contained 603 unique studies (avg. 22 per meta-analysis, range 2 to 108) and including a total 227,217 participants (average sample size per individual study within a meta-analysis 367, median 192, range 3 to 5354). A total of 1602 effect sizes were extracted and 145 originally reported summary effects.

### Prevalence of study type based upon sample size or pilot/feasibility classification

**Fig. 2:**
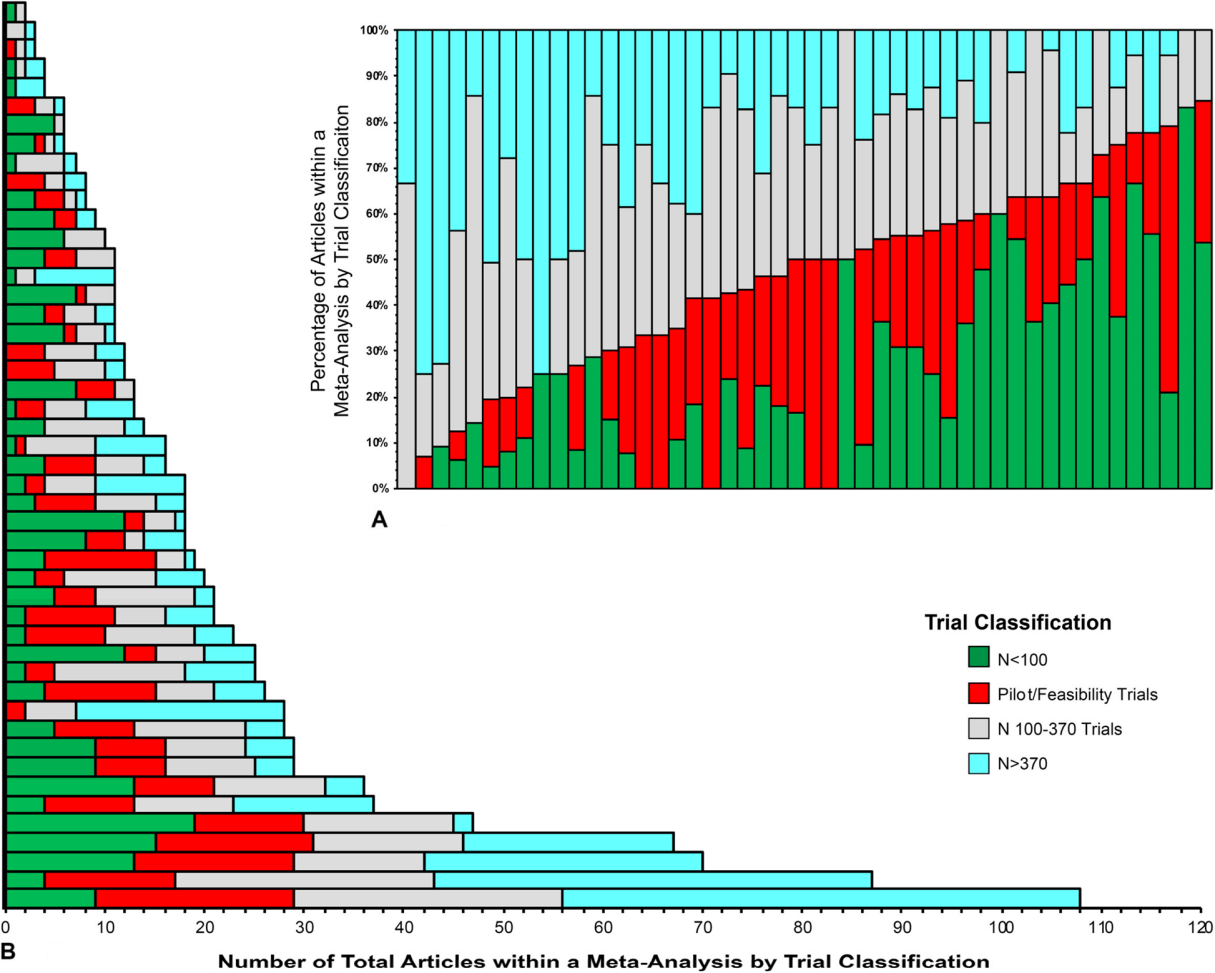
The number of studies included within each meta-analysis by trial classification. **A** The percentage of studies within each meta-analysis by trial classification. **B** The absolute number of studies by trial classification. Each bar represents one meta-analysis

The number and percentage of studies within a meta-analysis based upon the a priori classifications are depicted in Fig. 2. Based upon the a priori classifications of *N* ≤ 100 and pilot/feasibility trials, on average, these trials comprised 22% and 21% of all studies within the meta-analyses, respectively, with a combined prevalence of 39%. The largest prevalence of *N* ≤ 100 and pilot/feasibility trials within a given meta-analysis was 83% and 58%, respectively. Trials with *N* > 100 represented 57% of all articles. The upper 75th percentile in the distribution of the sample sizes was *N* > 370. The median sample size across classifications were 61 (IQR 34–121) for pilot/feasibility studies, 61 (IQR 40–78) for *N* ≤ 100 studies, 355 (range 102–1170) for *N* > 100 studies, and 740 (range 497–1170) for *N* > 370 studies. The summary effect size across all studies based upon pilot/feasibility and sample size classification were *N* ≤ 100 studies 0.40 (95% CI 0.35 to 0.45), pilot feasibility studies 0.25 (95% CI 0.20 to 0.30), *N* > 100 studies 0.17 (95% CI 0.14 to 0.21), and *N* > 370 0.12 (95% CI 0.08 to 0.17).

**Fig. 3:**
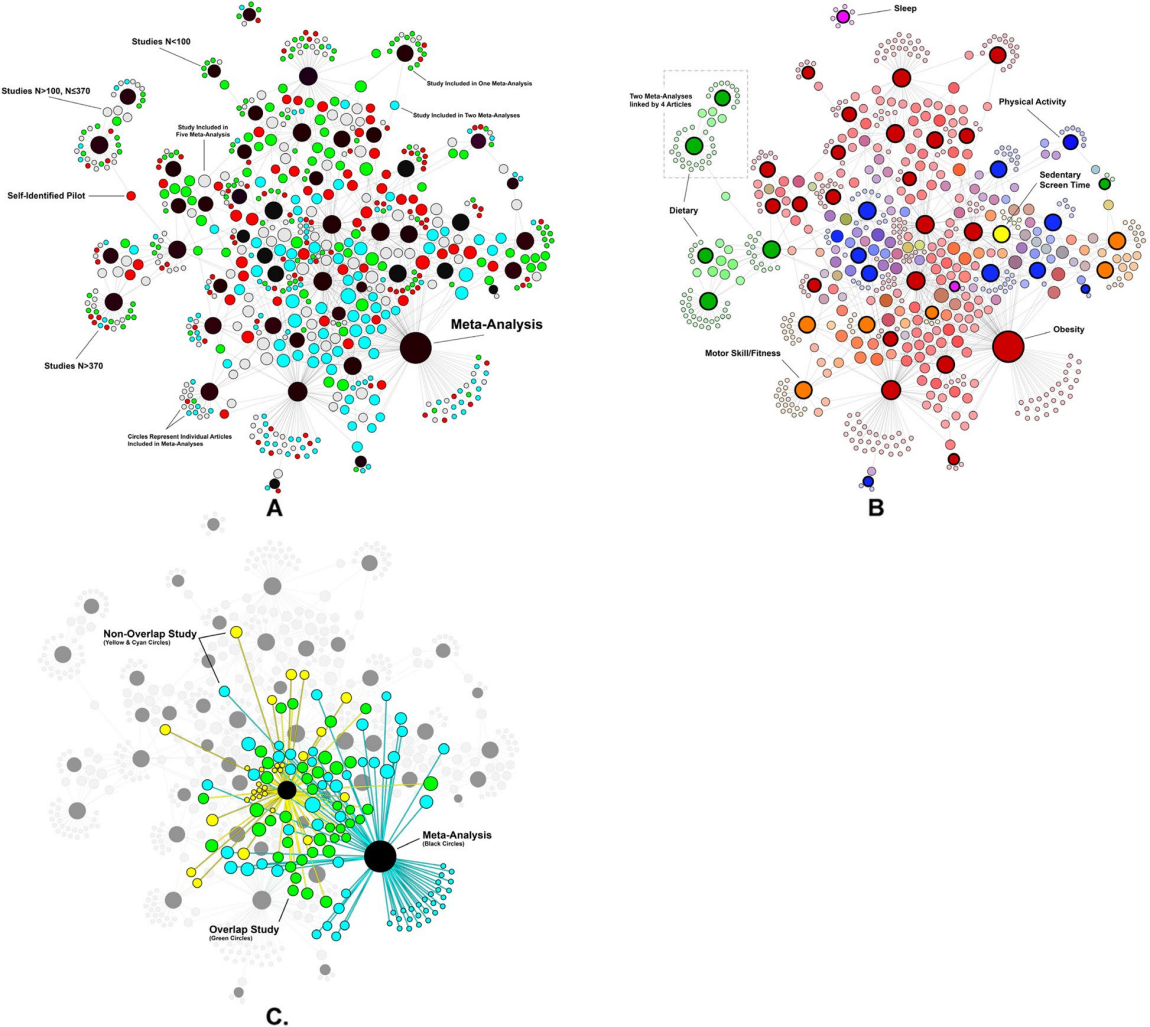
Network linkages between meta-analyses and the included studies based on trial classifications. **A** This network graph shows meta-analyses (black circles, nodes) and links with included studies (edges, for green, blue, red, and grey circles) based upon trial classification. The size of the circles represents either the number of studies included in the meta-analysis (for meta-analyses) or the number of times an article appears across different meta-analyses (for green, cyan, red, and grey circles). Red circles represent self-identified pilot studies; Green circles represent trials *N* ≤ 100; Grey circles represent trials *N* > 100 and *N* ≤ 370; and Cyan circles represent trials *N* > 370. **B** Network linkages between meta-analyses and included studies based on topic investigated in the meta-analyses. Red circles represent meta-analyses on a topic related to obesity; Green circles represent topics related to diet; Orange circles represent topics related to motor skill/fitness; Blue circles represent topics related to physical activity; Yellow circles represent topics related to sedentary/screen time behaviors. The size of the circles represents either the number of studies included in the meta-analysis or the number of times an article appears across different meta-analyses. **C** Example of network linkages between two meta-analyses and the overlap and non-overlap of the articles included. Yellow and cyan circles represent studies included within the meta-analysis that are not included in the other meta-analysis (non-overlap). Green circles represent studies included in both meta-analyses

The results of the network analysis are presented in Fig. 3. Studies classified as *N* ≤ 100 or pilot/feasibility trial were included in an average of 1.9 (range 1–6) and 2.5 (range 1–8) meta-analyses, respectively. Studies classified as *N* > 100 and *N* > 370 were included in an average of 2.9 (range 1–11) and 3.3 (range 1–11) meta-analyses. This resulted in an average study overlap of 29% within a meta-analysis, indicating that, on average, almost one-third of studies included in a meta-analysis were included in other meta-analyses on the same or similar topic. A single meta-analysis [26] consisted of articles with no (0%) overlap among articles included in the other meta-analyses, while all (100%) the studies included within one meta-analysis [19] were included in the other meta-analyses.

Table 1 presents the median and interquartile range for the originally estimated summary SMD, the re-estimated summary SMD, the absolute difference, and the percent difference in the estimates across study classifications for both the fixed and random effects models. Differences in the originally estimated effect size across comparisons is because not all of the included meta-analyses contained individual studies that covered all four of the mutually exclusive categories the studies were classified into. Across all comparisons, random effect models were more sensitive (i.e., demonstrated greater vibration) to the presence of trials of a given classification than fixed effect models. Removing either pilot/feasibility (median originally reported SMD 0.21 vs. median re-estimated SMD 0.22) or *N* ≤ 100 (0.24 vs. 0.16) resulted in a small difference between the originally reported SMD and the estimated SMD, with median absolute differences of 0.02 (IQR 0.01–0.05) and 0.04 (IQR 0.01–0.13), respectively. Restricting analyses to only *N* > 100 (0.21 vs. 0.12) or *N* > 370 (0.16 vs. 0.08) resulted in a modest median absolute difference of 0.05 (IQR 0.01–0.15) and 0.06 (IQR0.02–0.18). These translated into a median percent difference ranging from 13 to 52% across all four classifications.

**Table 1:** Comparison between originally reported meta-analytical effect sizes and the re-estimated effect sizes based on trial classifications

		Originally estimated summary standardized mean effect size^b^			Re-estimated summary standardized mean effect size			Absolute mean difference			Percent difference in standardized mean effect size		
Comparison	*k*	50th	(25th,	75th)	50th	(25th,	75th)	50th	(25th,	75th)	50th	(25th,	75th)
Random effects models													
* N* > 100^a^	113	0.21	(0.06,	0.40)	0.12	(0.05,	0.27)	0.05	(0.01,	0.15)	35%	(10%,	86%)
* N* > 370^a^	84	0.16	(0.05,	0.34)	0.08	(0.02,	0.18)	0.06	(0.02,	0.18)	52%	(20%,	130%)
Excluding pilot/feasibility	83	0.21	(0.06,	0.36)	0.22	(0.06,	0.35)	0.02	(0.01,	0.05)	13%	(6%,	38%)
Excluding *N* ≤ 100	83	0.24	(0.07,	0.40)	0.16	(0.06,	0.32)	0.04	(0.01,	0.13)	21%	(6%,	66%)
Fixed effects models													
* N* > 100^a^	113	0.14	(0.04,	0.28)	0.10	(0.03,	0.22)	0.02	(0.01,	0.06)	24%	(6%,	54%)
* N* > 370^a^	84	0.11	(0.03,	0.20)	0.07	(0.01,	0.15)	0.03	(0.01,	0.08)	31%	(8%,	79%)
Excluding pilot/feasibility	83	0.15	(0.04,	0.26)	0.14	(0.04,	0.26)	0.01	(0.00,	0.03)	11%	(4%,	27%)
Excluding *N* ≤ 100	83	0.16	(0.05,	0.29)	0.13	(0.05,	0.24)	0.02	(0.01,	0.05)	14%	(4%,	47%)

^a^Estimates exclude both pilot/feasibility and *N* ≤ 100 studies; *k* = number of summary effect sizes estimated

^b^Differences in the originally estimated effect size across comparisons is because not all of the included meta-analyses contained individual studies that covered all four of the mutually exclusive categories the studies were classified into

**Fig. 4:**
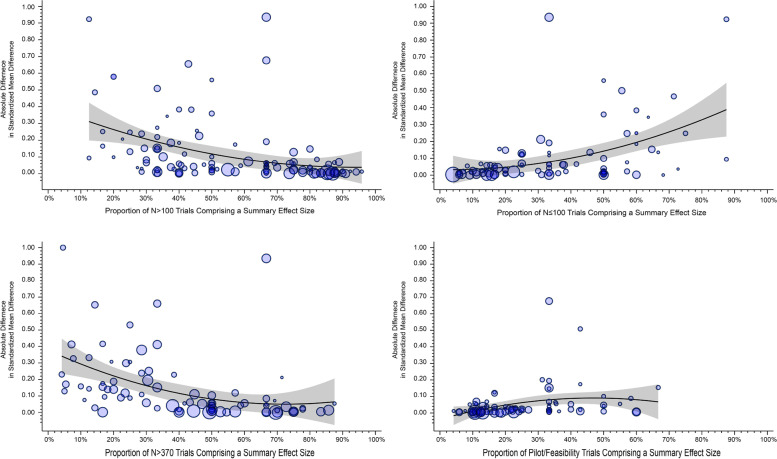
Influence of the proportion of trials, based on sample size or pilot/feasibility classification, within a meta-analysis on the absolute difference between the original estimated meta-analytical effect size and the re-estimated effect size using the random effects model. Size of the circle represents the weight in the meta-analysis

The association of the proportion of studies classified as *N* ≤ 100, pilot/feasibility, *N* > 100, and *N* > 370 included within a summary SMD on the absolute difference in the SMD based on the random effects models are presented in Table 2 and are depicted in Fig. 4. Across all four study classifications, as the proportion of studies comprising a summary SMD increased or decreased, it influenced the absolute difference between the originally reported summary SMD and the re-estimated summary SMD, with only the pilot/feasibility failing to reach statistical significance. When a summary SMD comprised > 60% of studies classified as *N* ≤ 100 this was associated with an absolute difference in the SMD of 0.29 (95% CI 0.16–0.41). Conversely, when summary SMDs comprised < 20% of studies classified as *N* > 100 or *N* > 370, the absolute difference in the SMD was 0.46 (95% CI 0.30–0.62) and 0.30 (95% CI 0.19–0.41), respectively.

**Table 2:** Meta-regression estimates of the association of the proportion of studies comprising a summary effect size based on trial classification

Trial classification	Independent variable	Estimate	SE	(95CI)	
Includes only studies with *N* > 100	Linear effect	− 0.31	0.08	(− 0.47,	− 0.15)
Excludes *N* ≤ 100 (i.e., small studies) and studies classified as pilot/feasibility	0–20%	0.46	0.08	(0.30,	0.62)
	20–40%	0.12	0.04	(0.04,	0.19)
	40–60%	0.13	0.04	(0.05,	0.21)
	60–80%	0.11	0.04	(0.04,	0.18)
	80–100%	0.02	0.04	(− 0.06,	0.10)
Includes only studies with sample sizes > 370	Linear effect	− 0.48	0.12	(− 0.71,	− 0.24)
Excludes *N* ≤ 370 and studies classified as pilot/feasibility	0–20%	0.30	0.06	(0.19,	0.41)
	20–40%	0.20	0.05	(0.09,	0.31)
	40–60%	0.04	0.05	(− 0.06,	0.15)
	60–80%	0.08	0.06	(− 0.04,	0.21)
	80–100%	0.01	0.15	(− 0.29,	0.32)
Includes studies with *N* > 100 and are classified as pilot/feasibility	Linear effect	0.39	0.10	(0.19,	0.59)
Excludes N ≤ 100 (i.e., small studies)	0–20%	0.03	0.03	(− 0.04,	0.09)
	20–40%	0.10	0.03	(0.04,	0.17)
	40–60%	0.11	0.04	(0.02,	0.19)
	60–100%	0.29	0.06	(0.16,	0.41)
Includes all studies except for studies classified as pilot/feasibility	Linear effect	0.16	0.09	(− 0.03,	0.34)
	0–20%	0.02	0.02	(− 0.02,	0.05)
	20–40%	0.08	0.02	(0.04,	0.12)
	40–60%	0.06	0.04	(− 0.02,	0.14)
	60–100%	0.02	0.06	(− 0.10,	0.13)

*Note*: Effects presented based on the comparison of the original standardized mean difference (SMD) and the re-estimated SMD using the random effects model

The concordance coefficient (kappa, κ) comparing the nominal level of significance (*P* < 0.05 vs. *P* > 0.05) between the originally estimated summary SMD and the re-estimated summary SMD based on study classifications are presented in Table 3. Excluding pilot/feasibility resulted in minimal influence on the concordance, with a κ 0.91 (0.89–0.94) for the fixed effects models and κ 0.81 (0.77–0.84) for the random effects models, whereas excluding *N* ≤ 100 studies reduced the concordance (κ 0.68, 0.64–0.72 fixed and κ 0.65, 0.60–0.69 random). Removing pilot/feasibility studies from meta-analyses rendered 4% and 8% of the significant effects non-significant and 14% and 17% non-significant with removal of *N* ≤ 100 studies. Removing both pilot/feasibility and *N* ≤ 100 studies by restricting analyses to only *N* > 100 and *N* > 370 studies resulted in a low concordance of κ 0.68 (0.63–0.72) fixed and κ 0.58 (0.53–0.62) random effect models for *N* > 100 and κ 0.47 (0.41–0.52) fixed and κ 0.35 (0.30–0.41) random for *N* > 370 studies. These estimates translate into 15% and 20% of the significant effects rendered non-significant when limited to *N* > 100 studies and 20% and 26% rendered non-significant when limited to *N* > 370 studies. Table 4 presents the association among study classification and precision. Across deciles, the least precise studies were mostly those classified as *N* ≤ 100 or pilot/feasibility studies and the least precise studies were associated with larger effect sizes. Restricting analyses to the upper 20% of precision, effect size estimates for *N* ≤ 100 and pilot/feasibility studies were 0.14 (95% CI 0.01 to 0.29) and 0.13 (95% CI 0.05 to 0.21), respectively, compared to 0.05 (95% CI 0.00 to 0.10) for *N* > 100 studies.

**Table 3:** Concordance between statistically significant (*P* < .05) originally reported meta-analytical effect size and the re-estimated effect size based upon trial classification

Fixed effects model							Random effects model				
	*k*	Kappa	(95CI)		Rendered non-significant	Rendered significant	*k*	Kappa	(95CI)	Rendered non-significant	Rendered significant
Trial classification											
*N* > 100^a^	113	0.68	(0.63,	0.72)	15%	0%	0.58	(0.53,	0.62)	20%	2%
*N* > 370^a^	84	0.47	(0.41,	0.52)	20%	5%	0.35	(0.30,	0.41)	26%	7%
Excluding pilot/feasibility	83	0.91	(0.89,	0.94)	4%	0%	0.81	(0.77,	0.84)	8%	1%
Excluding *N* ≤ 100	83	0.68	(0.64,	0.72)	14%	0%	0.65	(0.60,	0.69)	17%	1%

^a^Estimates exclude both pilot/feasibility and *N* ≤ 100 studies

**Table 4:** Comparison of precision (1/SE) and classification of studies based on pilot/feasibility or sample size categories

	Precision		Percentage of trials per decile of precision					
Decile of precision	Minimum	Maximum	*N* > 370	*N* > 100 to 369	*N* < 100	Pilot	ES	(95CI)
0 to 10%	1.11	2.95	1%	4%	36%	59%	0.44	(0.31, 0.56)
10 to 20%	2.96	3.75	0%	5%	51%	44%	0.58	(0.47, 0.69)
20 to 30%	3.78	4.36	2%	5%	67%	27%	0.32	(0.22, 0.43)
30 to 40%	4.36	5.32	4%	36%	40%	20%	0.32	(0.21, 0.42)
40 to 50%	5.33	6.57	5%	74%	7%	14%	0.26	(0.16, 0.36)
50 to 60%	6.58	8.05	3%	74%	4%	19%	0.20	(0.10, 0.30)
60 to 70%	8.06	9.83	29%	60%	2%	8%	0.16	(0.06, 0.26)
70 to 80%	9.83	11.57	77%	14%	1%	9%	0.17	(0.08, 0.27)
80 to 90%	11.59	15.76	78%	9%	4%	10%	0.08	(− 0.02, 0.18)
90 to 100%	15.81	106.50	88%	3%	3%	6%	0.04	(− 0.06, 0.13)

### Influence of study classifications on conclusions from meta-analyses: case study examples

**Fig. 5:**
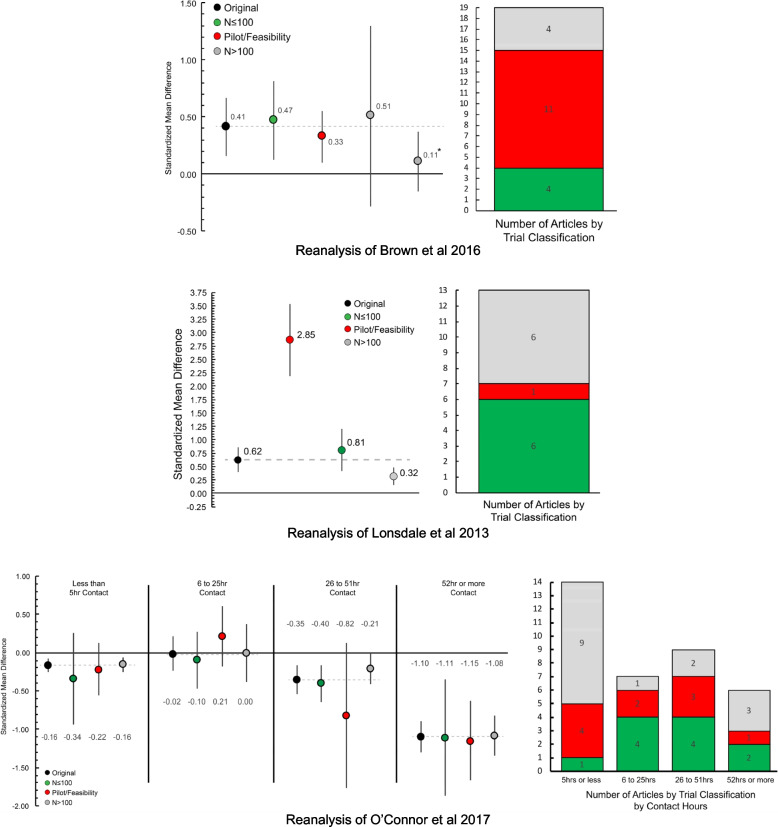
Case examples of reanalysis of meta-analyses considering trial classifications of three studies to inform the Community Preventive Services Task Force and the US Preventive Services Task Force. Dashed grey lines represent the standardized mean difference estimate originally reported in the publication. The “*” indicates sensitivity analysis, consistent with the original article’s analyses, where a single study was removed

Searching the Community Preventive Services Task Force and the US Preventive Services Task Force, we identified three meta-analyses that served as the basis for public health recommendations on a topic related to childhood obesity. The originally reported summary SMD and the re-estimated SMD restricted to the study classifications are presented in Fig. 5. For these analyses, we only considered *N* > 100 trials in the larger sample size categories given the limited number of studies in these meta-analyses with *N* > 370 (only 8 studies of a total of 68).

The first case study was the Brown et al. meta-analysis of family-based physical activity interventions for children (5–12 years) [22]. The conclusion drawn from the original meta-analysis findings were “…recommends interventions that actively engage families to increase physical activity by combining activities to build family support with health education. This finding is based on sufficient evidence of effectiveness in modestly increasing physical activity among children (pg 2) [65]”. The Brown et al. study [22] was comprised of 19 articles, of which 11 were self-identified pilot/feasibility trials (avg sample size 64 ± 33, range 27–132), 4 classified as *N* ≤ 100 trials (avg sample size 40 ± 17, range 29–67), and 4 as *N* > 100 (avg sample size 293 ± 146, range 117–441). Re-estimation of the SMD, considering only *N* > 100 studies resulted in a larger (0.51, 95% CI − 0.28 to 1.30) but non-significant SMD compared to the originally reported SMD (0.41, 95% CI 0.15 to 0.66). Consistent with the original analyses, we performed a sensitivity analysis with one study removed that substantially influenced the results; its removal reduced the re-estimated SMD restricted to *N* > 100 studies from 0.51 to 0.11 (95% CI − 0.15 to 0.37).

The second case study was the Lonsdale et al. meta-analysis of intervention to increase moderate-to-vigorous physical activity in physical education lessons. The recommendation drawn from the original meta-analysis findings was “…recommends enhanced school-based physical education to increase physical activity based on strong evidence of effectiveness in increasing the amount of time students spend in moderate- or vigorous-intensity physical activity during physical education classes (pg.2).” [66] The meta-analysis included 6 studies classified as *N* ≤ 100 (avg sample size 51 ± 20, range 26–81), one pilot/feasibility study (sample size 86), and 6 studies with *N* > 100 (avg sample size 484 ± 343, range 103–1048). The originally estimated summary SMD was 0.62 (0.40–0.86). The re-estimated summary SMD considering only pilot/feasibility or *N* ≤ 100 studies were 2.85 (2.18–3.53) and 0.81 (0.42–1.20). Restricting the re-estimation to only *N* > 100 studies rendered the summary SMD to 0.32 (0.16–0.47) or roughly half of the originally reported summary SMD.

The final case study was the O’Connor et al. [49] meta-analysis of intensive behavioral interventions to improve weight status of children (2–18 years). The recommendation drawn from the original meta-analysis findings was “Comprehensive, intensive behavioral interventions (≥ 26 contact hours) in children and adolescents 6 years and older who have obesity can result in improvements in weight status for up to 12 months; there is inadequate evidence regarding the effectiveness of less intensive interventions (pg. 2417).” [49, 67]. The re-analysis is restricted to behavioral interventions and reporting of weight change outcomes (i.e., BMI, zBMI). The O’Connor et al. study was comprised of 36 articles analyzed within four groups based upon the number of intervention contact hours. Of these, 11 were *N* ≤ 100 trials (avg sample size 69 ± 15, range 40–97), 10 pilot/feasibility trials (avg sample size 41 ± 21, range 15–70), and the remaining 15 *N* > 100 (avg sample size 264 ± 133, range 105–507). Re-estimation of the SMD for the 52 or more contact hours group resulted in no difference between the originally estimated SMD (− 1.10, 95% CI − 1.30 to − 0.89) and the restricted analysis considering only *N* > 100 studies (− 1.08, 95% CI − 1.34 to − 0.83). The re-estimated SMD for the 25–51 h contact group was rendered smaller and non-significant (− 0.21, 95% CI − 0.41 to 0.00) compared to the originally reported SMD for this group (− 0.35, 95% CI − 0.54 to − 0.16). No or minimal changes in the originally reported SMD for behavioral interventions comprising less than 5 h contact time (SMD − 0.16, 95% CI − 0.25 to − 0.08 vs. − 0.16, 95% CI − 0.25 to − 0.06) and those with 6–25 h (− 0.02, 95% CI − 0.24 to 0.21 vs. 0.00, 95% CI − 0.38 to 0.38) contact time was observed. The re-estimated SMD for the 26–51 h contact was similar to the originally reported and re-estimated SMD for the less than 5 h contact group (− 0.21 vs. − 0.16).

## Discussion

No single trial alone provides sufficient evidence to establish public health recommendations for behavioral interventions. Assembling information from multiple trials via systematic reviews and meta-analyses may represent the highest level of evidence upon which public health recommendations might be based. Conclusions from meta-analyses, however, may be misleading if they include a large share of studies that are pilot/feasibility and “small” trials because effects from such trials have been associated with inflated estimates of an intervention’s effects [5, 68, 69]. This study investigated the impact of including pilot/feasibility and small (i.e., *N* ≤ 100) studies on the summary effect size in meta-analyses of behavioral interventions in the area of childhood obesity. Overall, the exclusion of pilot/feasibility studies alone did not impact the re-estimated summary effect sizes, whereas excluding smaller studies (i.e., *N* ≤ 100) had a modest influence on the re-estimated effect sizes. Restricting analyses to only the relatively larger studies (i.e., *N* > 100 or *N* > 370) by excluding both pilot/feasibility and *N* ≤ 100 studies resulted in sizable differences in the re-estimated summary effect sizes, especially when 40% or fewer of the studies that comprised a summary effect sizes were either *N* > 100 or *N* > 370 studies. Thus, for meta-analyses where a large proportion of the studies that comprise the summary effect sizes are pilot/feasibility and small (i.e., *N* ≤ 100) studies, the estimated effect sizes are substantially different than the summary effect sizes restricted to the relatively larger studies (i.e., *N* > 100 or *N* > 370). Moreover, inclusion or not of the smaller studies may often change the presence or not of statistical significance.

These findings have important implications in the criteria for including studies in meta-analyses, particularly when the meta-analysis will likely be used to develop public health recommendations. Using the three identified meta-analyses that informed public health recommendations from the Community Preventive Services Task Force and the US Preventive Services Task Force, when removing both small and pilot/feasibility studies, the summary effect sizes were rendered non-significant or reduced in half in magnitude, leading to substantially different conclusions regarding the impact of the interventions reviewed. Restricting analyses to sample sizes of 100 or greater reduced the number of eligible studies markedly, with only 37% of the studies across all three meta-analyses meeting this sample size minimum. The reason why the majority of studies in these three meta-analyses and those included in the re-analyses presented herein are small or pilot/feasibility may reflect mostly the impact of convenience. Smaller and pilot/feasibility studies may require fewer resources and this could lead to a larger number of studies of these types being performed. Nevertheless, it is important to ask whether the effects estimated in smaller and pilot/feasibility studies are consistent with the expected effects when an intervention is widely endorsed and adopted [70–73].

As mentioned previously, both smaller and pilot/feasibility studies are associated with larger effects. Reasons for this are many. These include small and pilot/feasibility studies suffering from publication bias, where only larger effects from smaller studies are published. Smaller and pilot/feasibility studies often have methodological flaws, are shorter in duration, delivered by more highly specialized experts (rather than a lay community member), and can be delivered with more intense oversight than a larger study of the same or similar intervention [70, 74, 75]. Each of these can lead to larger effect sizes than what can be obtained in a larger-scale study. If these issues are present in such studies, the question is whether it is appropriate to include them in meta-analyses, particularly those meta-analyses that are used to inform public health recommendations. This is an important consideration that goes beyond statistical identification of small-study effects to whether studies of this type should be included in meta-analyses at all; and, if they are, what impact do they have on the estimated summary effect sizes and the conclusions drawn from them [76]. Others have suggested differentiating studies based upon precision (i.e., 1/standard error) or including only those studies that are the most precise (i.e., top 10% of precision) [9, 77] Smaller and pilot/feasibility studies are typically associated with less precision and this approach would lead to excluding a large number of studies of this type. As demonstrated in this study, both smaller and pilot/feasibility studies comprise the largest proportion of the least precise studies. However, considering only those smaller and pilot/feasibility studies that exhibited a high degree of precision (i.e., upper 20% of precision) their associated effect sizes were still 2 to 3 times greater than the larger studies of a similar precision. This indicates that using precision alone as a marker of studies to include in a meta-analysis may not fully rectify the underlying issues of smaller and pilot/feasibility studies that lead to effect sizes that are substantially greater than larger sample size studies. Hence, whether smaller and pilot/feasibility studies should be included in meta-analyses is a conceptual issue that cannot be resolved by computational processes alone.

One argument for the inclusion of smaller and pilot/feasibility studies is that all studies on a given topic are potentially relevant and represent the possible effect sizes for a given intervention [78]. It is recognized that not all studies are the same and will vary on key aspects, such as mean age of participants or dosage of the intervention received. In fact, attempting to identify two or more behavioral interventions that are identical on key aspects is likely futile. Collectively, if studies with known characteristics, such as smaller sample sizes or early stage pilot/feasibility, are consistently associated with inflated effect sizes [79], including them in meta-analyses becomes problematic. Recent studies indicate that when scaling smaller or pilot/feasibility studies to larger studies or moving from a more highly controlled to more pragmatic design, up to a 70% reduction in the overall effect size is observed [8, 70, 72, 73]. While smaller or pilot/feasibility studies play an important role in the development of behavioral interventions; as evidenced here, when combining them with larger sized studies (e.g., *N* > 100) for meta-analytical purposes, distortion of the summary effect sizes occurs, thereby leading to potentially spurious and over-optimistic conclusions regarding the strength of the associations. However, evidence-based decisions often rely upon the best evidence available not necessary the best evidence possible [1]. Certainly, we have to be careful on not letting the absence of great evidence get in the way examining good evidence. We also recognize there is no clear answer to how small is too small. The sample size cutoff used herein is largely arbitrary [6]. Even studies with several hundreds of participants are not necessarily large enough and certainly they are far from the prototype of mega-trials. Even the largest studies that we included in this meta-epidemiological assessment may have biased results. Regardless, evidence across multiple studies, including ours, indicates the smaller trials in a given field, on average, tend to demonstrate the largest effects [5]. This indicates that, at minimum, sensitivity analyses should be performed and presented that exclude the very small trials from the calculations.

There are several limitations in the current study. These include restricting the meta-analyses to only those investigating behavioral interventions related to childhood obesity and using cutoffs for sample size classification that were arbitrary (but pre-specified). Some of the individual studies were included in more than meta-analysis and we did not account for the duplication of this information in the summary effect sizes across the different meta-analyses. Given the level of analysis presented herein are at the summary effect level, and not based on individual studies, we believe the presence of duplicate study effects to be minimal. Moreover, not all meta-analyses have equal importance for public policy recommendations and similar changes in effect size estimates may affect some policy recommendations more than others.

## Conclusions

Summary effect sizes in meta-analysis that contain a substantial proportion of pilot/feasibility and small studies may be misleading in their overall magnitude and precision of the estimated effect sizes. Where meta-analyses contain 40% or fewer larger trials, sensitivity analyses should be presented that restrict summary effect sizes estimates to only the larger, non-pilot/feasibility studies. Meta-analyses conducted for public health recommendations should consider whether summary effect sizes based upon largely small and preliminary studies are likely to represent appropriately the anticipated effect sizes when a strategy is widely used in more real-world, pragmatic conditions.

## Supplementary Information

**Additional file 1. **Adapted PRIMSA 2009 Checklist.

**Additional file 2. **Search Strategy.

## Data Availability

The datasets used and/or analyzed during the current study are available from the corresponding author on reasonable request.

## Abbreviations

ES: Effect size
VoE: Vibration of effect sizes
PRISMA: Preferred Reporting Items for Systematic Reviews and Meta-Analyses
ABS: Absolute difference
SMD: Standardized mean differences
BMI: Body mass index
zBMI: BMI-for-age *z*-score
