# Supplementary material for: Influence of pilot and small trials in meta-analyses of behavioral interventions: a meta-epidemiological study
Source: Syst Rev. 2023 Feb 18;12:21. doi: 10.1186/s13643-023-02184-7 (PMC9938611; doi:10.1186/s13643-023-02184-7)
Supplement: Supplementary file 2 — Additional file 2. Search Strategy. [file 13643_2023_2184_MOESM2_ESM.docx]

Additional File 2: Search Strategy

Sample of search strategy used to identify reviews and systematic analyses investigating child obesity-related topics, restricted to articles published since January 1st, 2016

| PubMed  through 8/30/2019 | Web of Science  through 8/30/2019 | EBSCO  through 8/30/2019 | EMBASE  through 8/30/2019 |
| --- | --- | --- | --- |
| 1. meta-analysis  2. systematic  3. or/ 1-2  4. child*.ti,ab  5. adolescent$.ti,ab  6. infant$.ti,ab  7. youth$.ti,ab  8. preschool$.ti,ab  9. school$.ti,ab  10. student$.ti,ab  11. or/ 4-10  12. obesity.ti,ab  13. physical activ*.ti,ab  14. diet*.ti,ab  15. nutriti*.ti,ab  16. screen*.ti,ab  17. sleep*.ti,ab  18. exercis*.ti,ab  19. fitness.ti,ab  20. sport$.ti,ab  21. overweight.ti,ab  22. or/ 4-12  23. 3 AND 11 AND 22 | 1. meta-analysis  2. systematic  3. or/ 1-2  4. child*.ti,ab  5. adolescent$.ti,ab  6. infant$.ti,ab  7. youth$.ti,ab  8. preschool$.ti,ab  9. school$.ti,ab  10. student$.ti,ab  11. or/ 4-10  12. obesity.ti,ab  13. physical activ*.ti,ab  14. diet*.ti,ab  15. nutriti*.ti,ab  16. screen*.ti,ab  17. sleep*.ti,ab  18. exercis*.ti,ab  19. fitness.ti,ab  20. sport$.ti,ab  21. overweight.ti,ab  22. or/ 4-12  23. . 3 AND 11 AND 22 | 1. meta-analysis  2. systematic  3. or/ 1-2  4. child*.ti,ab  5. adolescent#.ti,ab  6. infant#.ti,ab  7. youth#.ti,ab  8. preschool#.ti,ab  9. school#.ti,ab  10. student#.ti,ab  11. or/ 4-10  12. obesity.ti,ab  13. physical activ*.ti,ab  14. diet*.ti,ab  15. nutriti*.ti,ab  16. screen*.ti,ab  17. sleep*.ti,ab  18. exercis*.ti,ab  19. fitness.ti,ab  20. sport#.ti,ab  21. overweight.ti,ab  22. or/ 4-12  23. . 3 AND 11 AND 22 | 1. meta-analysis  2. systematic  3. or/ 1-2  4. child*.ti,ab  5. adolescent$.ti,ab  6. infant$.ti,ab  7. youth$.ti,ab  8. preschool$.ti,ab  9. school$.ti,ab  10. student$.ti,ab  11. or/ 4-10  12. obesity.ti,ab  13. physical activ*.ti,ab  14. diet*.ti,ab  15. nutriti*.ti,ab  16. screen*.ti,ab  17. sleep*.ti,ab  18. exercis*.ti,ab  19. fitness.ti,ab  20. sport$.ti,ab  21. overweight.ti,ab  22. or/ 4-12  23. . 3 AND 11 AND 22 |
